# Horizontally Elongated Time Domain Reflectometry System for Evaluation of Soil Moisture Distribution

**DOI:** 10.3390/s20236834

**Published:** 2020-11-29

**Authors:** Dong-Ju Kim, Jung-Doung Yu, Yong-Hoon Byun

**Affiliations:** 1 School of Agricultural Civil & Bio-Industrial Engineering, Kyungpook National University, Daegu 41566, Korea; kyrix1028@knu.ac.kr; 2 School of Civil, Environmental and Architectural Engineering, Korea University, Seoul 02841, Korea; noorung2@korea.ac.kr

**Keywords:** elongated probe, moisture distribution, soil water content, time domain reflectometry

## Abstract

The objective of this study was to develop a horizontally elongated time domain reflectometry (HETDR) system to evaluate the water content in nonuniformly wetted soils. The HETDR probe consists of three rods of stainless steel and a cuboid head: A center electrode and two outer electrodes are connected to the inner and outer conductors of a coaxial cable, respectively. An acrylic container divided into several segments was used to prepare nonuniformly wetted soils with different water contents for a series of model tests. The HETDR probe was placed horizontally at the middle height of each soil specimen, while a conventional time domain reflectometry (TDR) probe was applied vertically on the surface of the specimen. The experimental results show that as the soil water content (SWC) at a segment increases, the average amplitude decreases and the duration increases. The estimated SWC increases with the measured SWC, and especially, the difference between actual segment length and the segment length estimated from the HETDR probes is significant under dry conditions. This study demonstrates that HETDR may be a promising field-testing method for evaluating the average water content in nonuniformly wetted soils.

## 1. Introduction

Climate change affects countries all over the world; hence, an efficient irrigation system is essential for the proper management of soil moisture in the root zone of crops, since this factor heavily influences their growth [1,2]. Accordingly, it is necessary to establish an adequate method for measuring soil moisture content and, hence, ensure an efficient water resource management.

Soil water content (SWC) data have been previously obtained mainly by gravimetric sampling and with the use of tensiometers, which could provide information on water content distribution [3,4,5]. Tensiometers have been widely used because of their low cost and simple installation; however, due to their operating principle, the measurement range of tensiometers is limited to a certain value of negative pressure generated by soil moisture [6,7]. The electrical conductivity method has also been used to estimate the water content: The electrical conductivity of soils depends on their water content [8]. Recently, electromagnetic wave-based techniques (e.g., time domain reflectometry (TDR), frequency domain reflectometry, capacitive sensors, and visible and near infrared spectroscopy) have been developed [9,10]. Among these, TDR is the most common method for determining the SWC. This method is based on the determination of soil permittivity and the application of an empirical relationship [11,12,13]. All of the above methods are usually point-based, and the necessary instruments are vertically applied on soils: Characterizing the horizontal distribution of the SWC in cultivated fields requires the use of numerous sensors [14].

Various types of TDR probes have been applied to measure the SWC. For example, a two-rod TDR probe with an impedance-matching transformer was introduced by Davis [15], while a two-rod TDR probe without transformer was used by other researchers [16,17,18]. Additionally, a three-rod TDR probe was proposed by Zegelin et al. [19]. This does not require any transformer and usually provides simpler waveforms and more distinct reflections compared to that of two-rod TDR probes. Selker et al. [20] developed a flat and rectangular TDR probe for the measurement of soil surface water content. Penetration-type TDRs were used to reconstruct profiles of volumetric water content in shallow-depth soils [21,22,23]. Notably, Topp et al. [24] applied the TDR method with parallel wire transmission lines for monitoring the progression of a wetting front through soil. Miyamoto et al. [25] suggested the application of multiple length TDR probes to measure vertical water distributions in soil. Despite these previous efforts, the application of TDR probes to nonuniformly wetted soils in the horizontal direction is still needed for a complete estimation of soil moisture distribution.

The TDR method, which is based on the electromagnetic response of the soil, has been applied in geotechnical engineering; in particular, it was used for the nondestructive monitoring of soil nails, evaluating their installed length and grouted length [26]. Lee et al. [27] reported that the TDR is an effective method for detecting necking defects in bored piles. Another application of the TDR method is the identification of defects in rock bolts used to improve the stability of tunnels and rock slopes [28]. Furthermore, Yu et al. [29] suggested the use of a circular TDR system to enhance the resolution of scour depth monitoring around piers and bridge abutments. Only a few studies have successfully assessed the effect of nonuniform moisture distribution on TDR waveforms and the reliability of the water content measurements averaged through nonuniformly wetted soils.

The purpose of this study was to develop a horizontally elongated TDR and evaluate the variability of the TDR response, the potential for the estimation of heterogeneous SWC distribution, and the reliability of the average water content obtained for nonuniformly wetted soils. Here, the theoretical background of the TDR method is explained and the concept of horizontally elongated time domain reflectometry (HETDR) with a different probe length is introduced; moreover, the experimental setup considered for the calibration and model tests is presented. The calibration results were interpreted to explain the effects of probe length and spacing on the calibration curve; additionally, based on the results of the model test, it was possible to analyze the effects of water content distribution on the TDR waveforms. The performances of HETDR probes having different lengths are discussed by comparing them with those of conventional TDR probes.

## 2. Materials and Methods

### 2.1. Theoretical Background

The TDR method (based on the transmission line theory) was used to measure the water content of a specific soil layer around the TDR probe. This method is based on the concept that the propagation velocity of an electromagnetic wave (*v*) along the embedded probe length (*L*) is a function of the speed of light (*c*) and of the dielectric constant of a low-loss medium (*K*):

(1) $$v=\frac{c}{\sqrt{K}}$$

When the length of the probe used for the transmission of electromagnetic waves is known, the wave velocity can be determined from the travel time (*t*):

(2) $$v=\frac{2L}{t}$$

Note that the probe length is doubled since the transmitted wave is reflected at the tip of the probe and recorded by the reflectometer. Using Equations (1) and (2), an apparent dielectric constant (*K_a_*) was defined as follows:

(3) $$K_{a}={\left(\frac{ct}{2L}\right)}^{2}$$

When the material is uniform and lossless, the apparent dielectric constant is approximately equal to the true material dielectric constant (*K*). In layered media, the electromagnetic waves measured by a TDR system are reflected by interfaces, resulting in electrical impedance differences. Yu et al. [29] showed that the water level and scour depth can be determined from TDR responses.

### 2.2. HETDR

**Figure 1:**
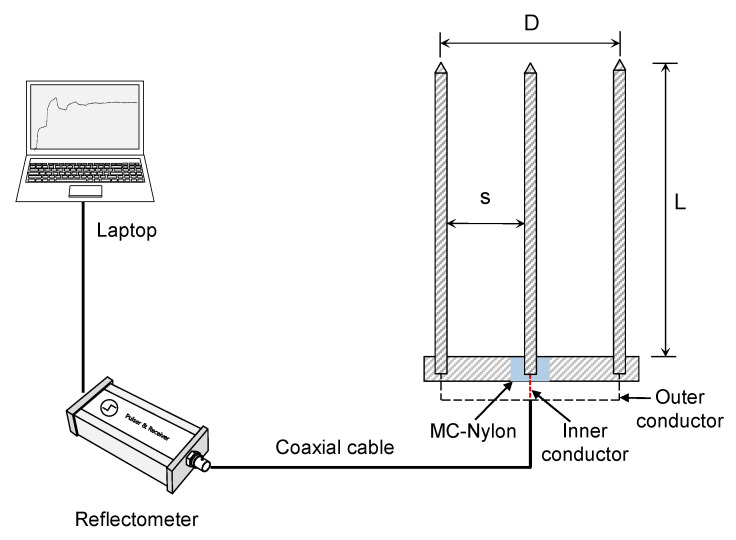
Schematic drawing of the horizontally elongated time domain reflectometry (HETDR) system. s, D, and L indicate the probe spacing, probe width, and probe length, respectively.

Generally, two-rod TDR probes have larger sample areas compared to that of three-rod ones having the same rod spacing (*s*) and rod diameters (*d*). In this study, a three-rod probe was selected for the HETDR system in order to avoid the need for an impedance-matching transformer. Usually, three-rod probes provide simpler waveforms and more distinct reflections compared to those of two-rod probes [30]. Figure 1 shows the three rods of the probe selected for the HETDR system, each having a diameter of 0.6 cm. These rods, made of stainless steel, were mounted on a cuboid head with a length of 16.5 cm, a height of 2 cm, and a depth of 1.5 cm by fastening a screw at the end of each rod. An insulation cap of mono cast nylon was placed between a center electrode and the cuboid head. The center electrode and two outer electrodes were connected to the inner and outer conductors of a 50-Ω coaxial cable, respectively. Three different probe lengths (ranging from 50 to 100 cm) were used in this study; moreover, the spacing of the electrodes on each cuboid head was adjustable (Table 1). The ratio between the diameter and the spacing of the rods (*d/s*) ranged from 0.08 to 0.24. Knight [31] suggested that this ratio should be greater than 0.1 to ensure a reduction of the skin effect (the tendency of the total energy per unit length to be concentrated within a cylindrical region). In contrast, Petersen et al. [32] reported that the volumetric water content can be appropriately determined with a configuration as small as *d/s* of 0.02. The TDR unit (HL1101, Hyperlabs) was used to generate a step pulse with an input voltage of 250 mV and to record the reflected signals. Overall, 25 signals were stacked to increase the signal-to-noise ratio.

### 2.3. Calibration

**Figure 2:**
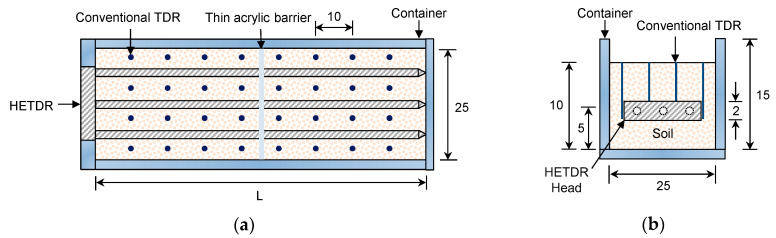
HETDR probe with a container: (**a**) plan view; (**b**) side view. All values are expressed in cm. TDR: time domain reflectometry.

The empirical relationship between volumetric water content and apparent dielectric constant suggested by Topp et al. [12] has been widely used to evaluate water content in the field using the conventional TDR method. HETDR probes need to be calibrated for their use in soils. In this study, an acrylic container with a width of 25 cm and a height of 15 cm was used for the calibration of several HETDR probes, as shown in Figure 2. The length of the container could be changed according to the length of each HETDR probe. A sandy soil with a mean diameter of 0.56 mm and a coefficient of uniformity of 1.46 was poured in the container, up to the height of 10 cm. Then, the HETDR probes were placed horizontally at the middle height of each soil specimen. The calibration of the HETDR probes was carried out in homogeneous soils with 10 different water contents (ranging from 0 to 0.38 m^3^ m^−3^). After measuring the electromagnetic waves using the HETDR probes, conventional TDR probes were also applied vertically at 10 cm intervals around the HETDR probes to compare the respective water content measurements (Figure 2a). To evaluate the effects of probe spacing on the calibration curve, HETDR probes with three different probe lengths and spacings were used for the calibration.

### 2.4. Model Test

The acrylic container used for the calibration of the HETDR probes was divided into several different segments. HETDR probes with different lengths (50 and 100 cm) but with the same spacing (15 cm) were used for evaluating the water content distribution in the soil inside the container, as summarized in Table 2. Four different treatments of water content distribution were selected; moreover, for a treatment, the SWC in each segment was changed. A thin acrylic barrier with three holes was designed to maintain the desired SWC in each segment and allow the passage of electrodes through the barrier itself. This acrylic barrier was applied to the interface between two segments, assuming that it would rarely influence the TDR response. Note that the dielectric constant of the acrylic material was similar to that of dry soils. Except for the barrier, the test condition and procedure considered for the model tests were similar to those used for the calibration. After preparing the soils in the segments, the TDR responses were recorded by using the HETDR system. After the measurement of the electromagnetic waves by using the HETDR probes, the conventional TDR probes were applied at 10 cm intervals around the HETDR probes to compare the water content values measured by the two types of instruments.

## 3. Results

### 3.1. Probe Length and Spacing

**Figure 3:**
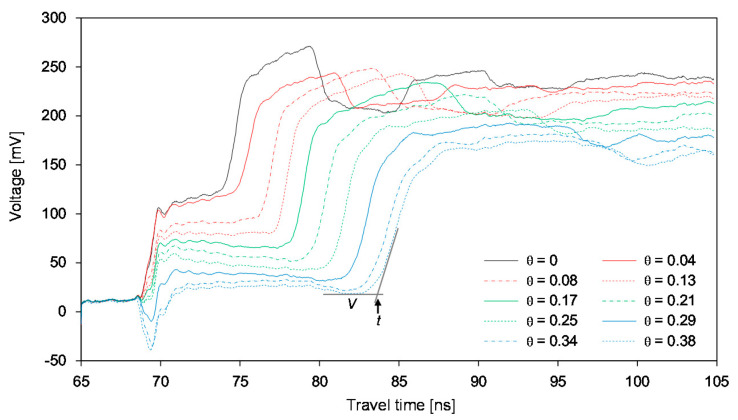
Variance of the signals in the uniform soil with water content obtained by using a probe with a length of 50 cm. θ denotes the SWC.

**Figure 4:**
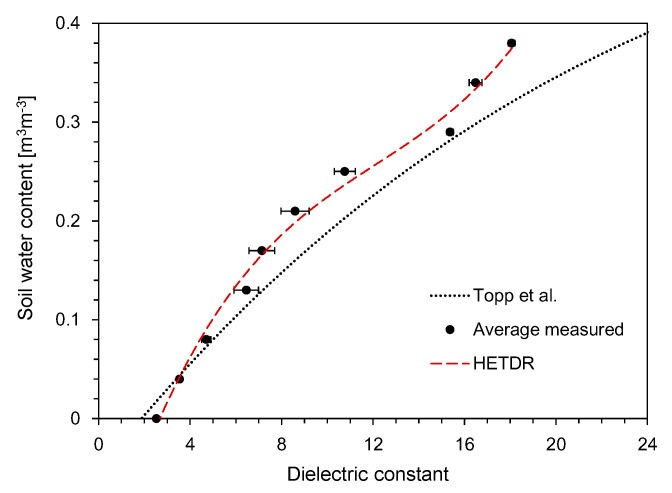
Relationships between the SWC and the dielectric constant for different probe spacings.

The typical waveforms obtained from the HETDR probe with a length of 50 cm are plotted in Figure 3. With the increase of the SWC, the signal amplitude (*V*) after partial reflection from the beginning of the probe decreased and the travel time (*t*) of the signal in the probe increased. For the three different probe lengths with the same probe spacing, the travel time was found to increase with the probe lengths, while their signal amplitudes were similar. The apparent dielectric constant of each probe was determined from the travel time of the signals and Equation (3). Figure 4 shows the relationship between the dielectric constant and the water content measured by the probes having lengths of 50 cm and three different spacings. In general, the average dielectric constants determined by HETDR increased with the water content, reflecting a third degree polynomial relationship. This relationship was well established, and its coefficient of determination was equal to 0.988. Notably, the *d/s* values obtained for probe spacings of 2.5 and 5.0 cm were greater than those recommended by Knight [31] to reduce the energy concentration around the rods of a probe. The standard deviations for SWC were between 0.13 and 0.25 m^3^ m^−3^ and were significant compared to those for lower and higher SWC vaules. For instance, the dielectric constants determined by HETDR for SWC > 0.04 m^3^ m^−3^ have values lower than those determined from the empirical relationship suggested by Topp et al. [12].

**Figure 5:**
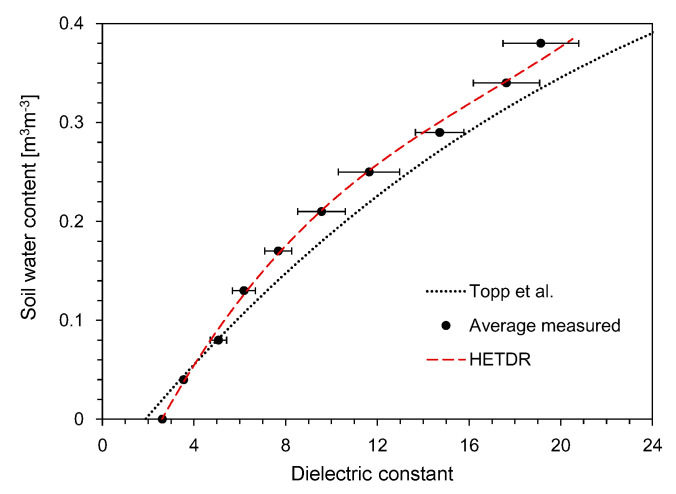
Relationships between the SWC and the dielectric constant for different probe lengths.

Figure 5 shows the relationship between the dielectric constant and the SWC for probes having a constant spacing of 7.5 cm and three different lengths. The average values of the dielectric constants determined by HETDR increased with the water content, reflecting a third degree polynomial relationship (coefficient of determination = 0.983). Although the standard deviations for higher SWC were greater than those for lower SWC, the third degree polynomial relationships established for all probes had coefficients of determination >0.994.

### 3.2. TDR Waveforms

**Figure 6:**
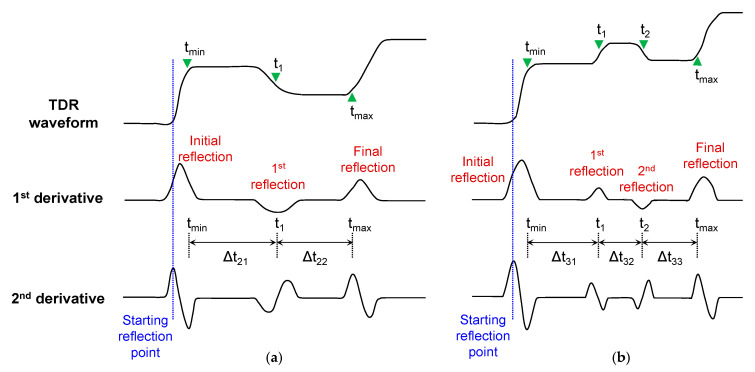
Typical TDR waveforms and the variation of first and second order derivatives in: (**a**) two segments (DW); (**b**) three segments (WDW).

Four different model tests were carried out to evaluate the effect of water content distribution on waveforms using HETDR probes with a width of 15 cm and two different lengths (0.5 and 1 m). For the model tests, the SWC was changed in each segment. Typical waveforms recorded in the soil containers divided into two and three segments are plotted in Figure 6. The first and second order derivatives of the waveform travelled with respect to time. In this study, the starting reflection point is defined as the time of maximum second order derivative in the initial reflection, and additionally, the time of minimum second order derivative in the first reflection (*t_min_*) is used. The end reflection point is defined as the time of maximum second order derivative in the final reflection (*t_max_*). For the nonuniformly wetted soils prepared in the two and three segments, one or two additional reflections between the initial and final reflections occured. The inflection points in the additional reflections are determined from the time (*t*_1_ and *t*_2_) of maximum or minimum first order derivative in the reflections. Note that, in practice, the maximum and minimum second order derivatives in the additional reflections are not often clarified, and thus, the time of maximum and minimum first order derivatives in the reflections were used in this study. The durations *Δt*_21_ (= *t*_1_ − *t_min_*) and *Δt*_22_ (= *t_max_* − *t*_1_) were used for analyzing the TDR waveforms obtained in two segments. For three segments, the durations *Δt*_31_ (= *t*_1_ − *t_min_*), *Δt*_32_ (= *t*_2_ − *t*_1_), and *Δt*_33_ (= *t_max_* − *t*_2_) were used. 

**Figure 7:**
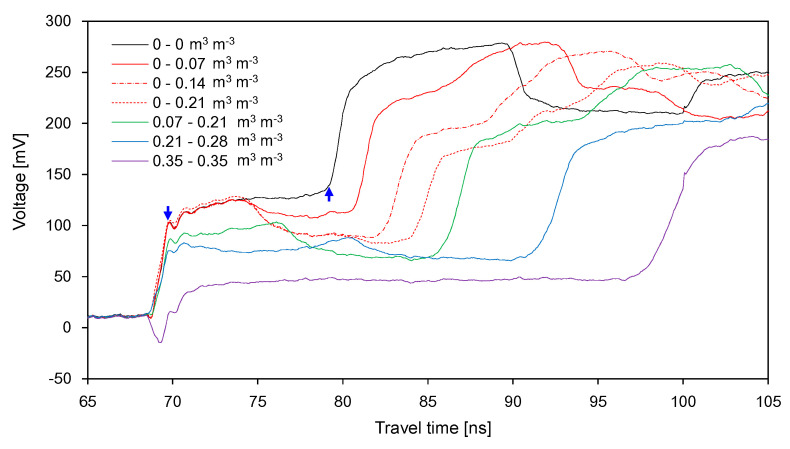
Variance of the signals with the SWC in the dry-wet treatment. The upward and downward arrows denote the end reflection point (*t_max_*) and the time of minimum second order derivative in the first reflection (*t_min_*), respectively.

**Figure 8:**
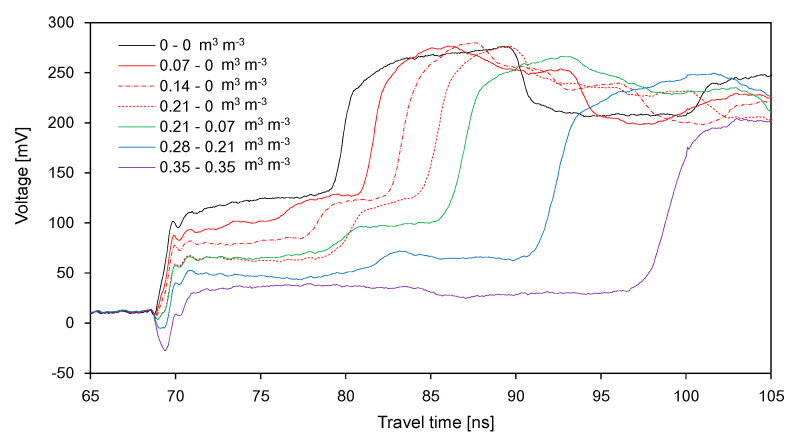
Variance of the signals with the SWC in the wet-dry treatment.

The waveforms recorded in the soil container divided into two segments are plotted in Figure 7 and Figure 8. Figure 7 shows the waveforms obtained from the dry-wet treatment (DW). Under dry conditions, the signal amplitude rapidly increased from the starting point and then converged to a certain value up to the end reflection point. However, an additional reflection occurred under partially wet conditions. The signal amplitudes before the first reflection under the first three partially wet conditions were same under dry conditions: The soils in the first segment remained dry. The average amplitude for *Δt*_22_ decreased with the increase in SWC in the second segment; moreover, *Δt*_22_ increased with the increase in SWC in the second segment. As the SWC increased in the first segment, the average amplitude for *Δt*_21_ decreased, whereas *Δt*_21_ increased. Considering that the electrical conductivity of the soil can be represented by the signal amplitudes [33], the variation in the average amplitude for each duration depends on the electrical conductivity of the soil. As the SWCs in the two segments were closer together, the difference between the average amplitudes for *Δt*_21_ and *Δt*_22_ decreased. Overall, the total travel time, which is the sum of *Δt*_21_ and *Δt*_21_, increased with the average SWC in the container.

The waveforms obtained from the wet-dry treatment (WD) are plotted in Figure 8. Under the first three partially wet conditions (indicated by red lines), the average amplitude for *Δt*_21_ decreased with increasing SWC in the first segment, and *Δt*_21_ increased with increasing SWC in the first segment. Under the same conditions, the average amplitude for *Δt*_22_ remained almost constant, because the soils in the second segment remained dry. Similar to the DW treatment, the total travel time increased with increasing average SWC in the container. In contrast to the results of the DW treatment, according to the SWC in each segment, the average amplitude for *Δt*_22_ was greater than that for *Δt*_21_. For average SWC values of 0.105 to 0.175 m^3^ m^−3^, *Δt*_21_ and the average amplitude for *Δt*_21_ remained almost constant, whereas the average amplitude for *Δt*_22_ decreased with an increase in the average SWC. Note that, for average SWC values of 0.105 to 0.175 m^3^ m^−3^, the SWC in the first segment did not vary, whereas the SWC in the second segment increased. Overall, *Δt*_21_, *Δt*_22_, and the average amplitudes for *Δt*_21_ and *Δt*_22_ varied with the SWC in each segment. Under dry and saturated conditions, identical waveforms were obtained from the DW and WD treatments. The SWC of 0.35 m^3^ m^−3^ represents that the soil may be under the saturated condition.

**Figure 9:**
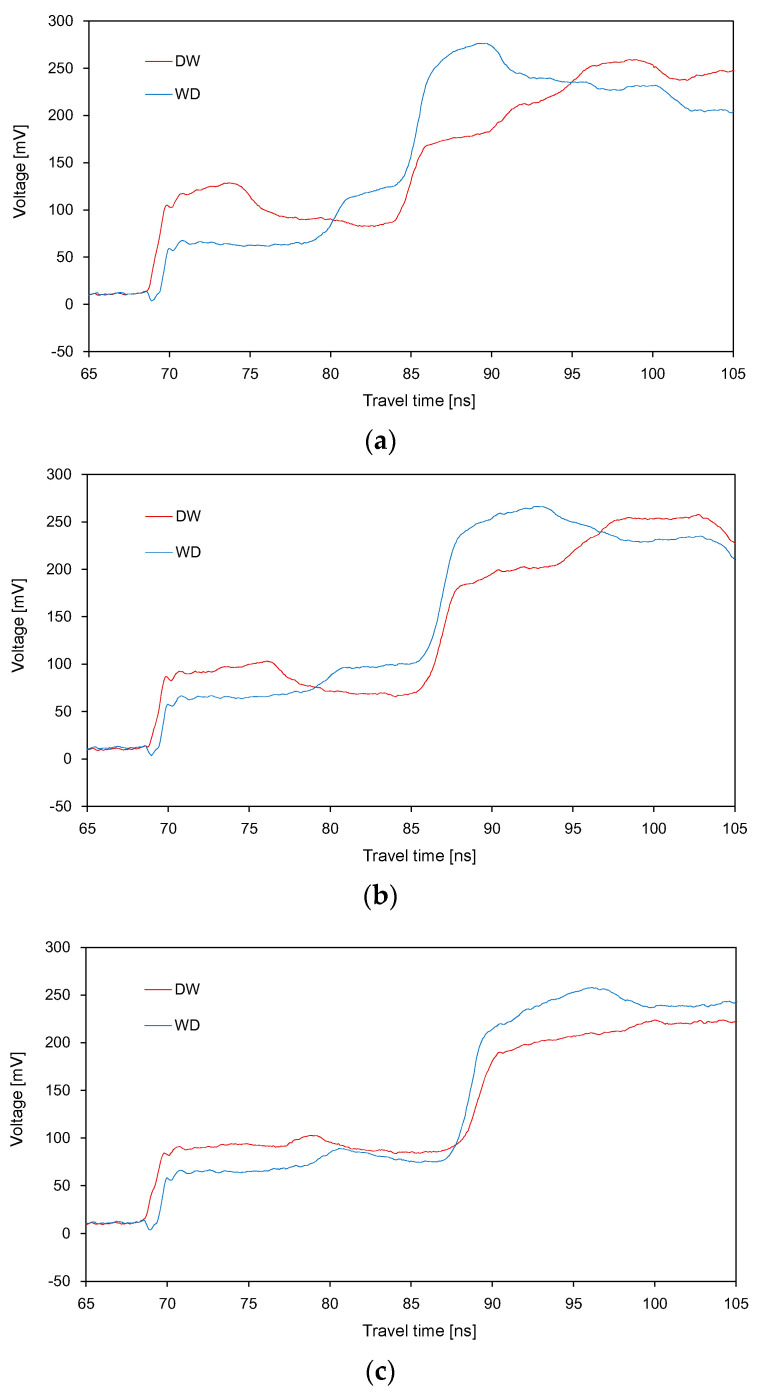
Comparison between the signals obtained from the two-segmented containers at average SWCs of: (**a**) 0.105; (**b**) 0.14; (**c**) 0.175.

The waveforms obtained for three different average SWC values (0.105, 0.14 and 0.175 m^3^ m^−3^) through the DW and WD treatments are plotted in Figure 9. For each average SWC value, the waveforms obtained from the two segments in a different sequence were compared. When the difference in SWC between the two segments was significant, the difference in the average amplitudes for *Δt*_21_ and *Δt*_22_ between the DW and WD treatments was also significant (Figure 9a). Moreover, Figure 9b,c show that, as the difference in SWC between the two segments decreased, the difference in the average amplitudes for *Δt*_21_ and *Δt*_22_ between the DW and WD treatments also decreased. For the same average SWC, the values of total travel time obtained from the DW and WD treatments were similar, regardless of the different sequences.

**Figure 10:**
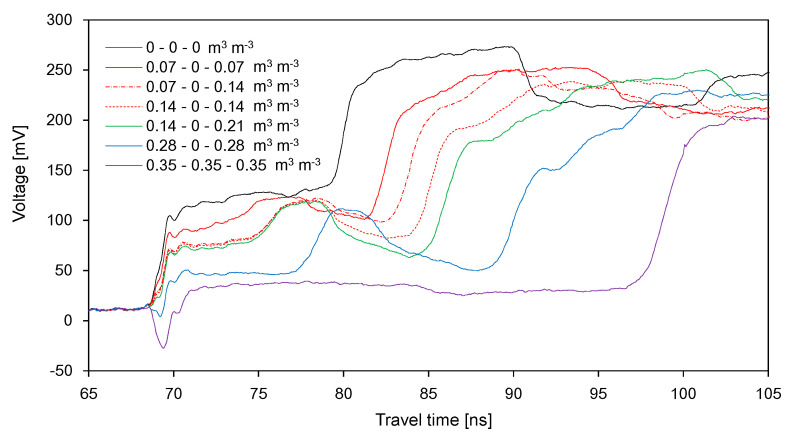
Variance of the signals with the SWC in the wet-dry-wet treatment.

The waveforms in the soil container divided into three segments and obtained from the wet-dry-wet (WDW) treatment are plotted in Figure 10. Similarly to the WD treatment, as the SWC in the first segment increased, the average amplitudes for *Δt*_31_ gradually decreased and *Δt*_31_ increased. In the case of the WDW treatment, moreover, the average amplitudes for *Δt*_32_ rarely changed with an increase in the average SWC, because the soils in the second segment remained dry. As the SWC in the third segment increased, *Δt*_33_ increased, whereas the average amplitudes for *Δt*_33_ decreased.

**Figure 11:**
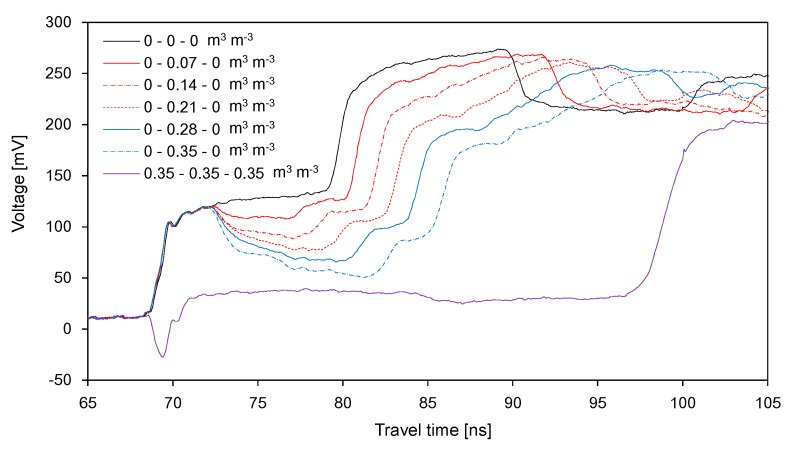
Variance of the signals with the SWC in the dry-wet-dry treatment.

Figure 11 shows the waveforms obtained from the dry-wet-dry (DWD) treatment. In this treatment, the soils in the first and third segments remained dry, while the SWC in the second segment varied. Accordingly, as the average SWC increased up to 0.117 m^3^ m^−3^, *Δt*_31_ and the average amplitude for *Δt*_31_ remained constant, while the average amplitudes for *Δt*_32_ and *Δt*_33_ decreased; in addition, *Δt*_32_ increased with the average SWC.

## 4. Analyses

### 4.1. Estimation of Segment Length and Water Content

**Figure 12:**
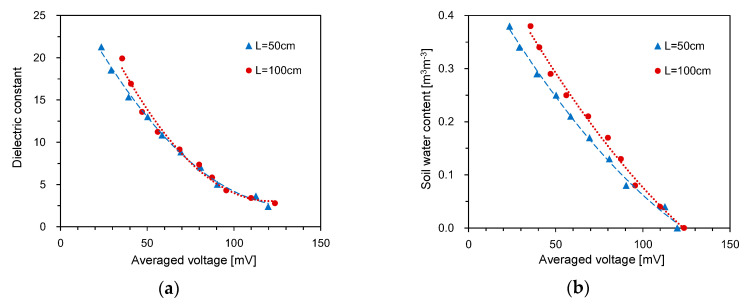
Relationships between average amplitude and: (**a**) dielectric constant; (**b**) SWC.

The electrical conductivity of soils has been determined from the signal amplitude of the TDR waveform [19,33,34,35,36,37]. A number of studies represented that the electrical conductivity of soil pore water is related to the water content values and the electrical conductivities of soils [38,39,40,41,42]. Assuming that the electrical conductivity of soil pore water remains constant, the electrical conductivity of soils can be related with the SWC. In this study, the average amplitude for each duration was used to estimate the SWC in each segment. First, the average amplitude and the dielectric constants determined from the TDR waveforms in the homogeneous soils with 10 different water content values are plotted in Figure 12a. For both probe lengths, the dielectric constant decreased with an increase in the average amplitude along a quadratic polynomial function. Figure 12b shows the relationships between the measured SWC and the average amplitude. The SWC decreased with an increase in the average amplitude along the quadratic polynomial function. 

The relationships of the average amplitude (*V_a_*) with the dielectric constant (*K_a_*) and SWC (*θ*) can be represented as follows:

(4) $${\left(K_{a}\right)}_{ij}=A{\left(V_{a}\right)}_{ij}{}^{2}-B{\left(V_{a}\right)}_{ij}+C$$

(5) $$\theta_{ij}=D{\left(V_{a}\right)}_{ij}{}^{2}-E{\left(V_{a}\right)}_{ij}+F$$

where *A*, *B*, and *C* are the model parameters for estimating the dielectric constant. *D*, *E*, and *F* are the model parameters for estimating the SWC. The subscripts of *i* and *j* indicate the number of segments and the order of the segments, respectively. The values of the model parameters and coefficient of determination are summarized in Table 3. The relationships between the average amplitude and the dielectric constant for both probe lengths were almost identical. At the same SWC, the average amplitudes for the probe length of 100 cm were greater than those for the probe length of 50 cm. All relationships showed the coefficient of determination to be higher than 0.986. 

**Figure 13:**
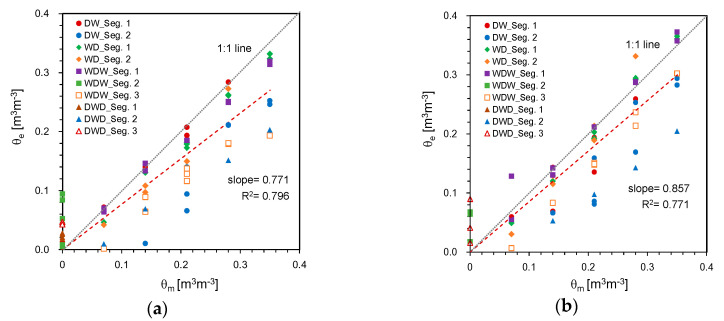
Comparison of SWCs measured and estimated with the probe lengths of: (**a**) 50 cm; (**b**) 100 cm. θ_m_ and θ_e_ denote the SWCs measured and estimated from HETDR, respectively.

Using the average amplitude for each duration in the four treatments and Equation (5), the SWC for each duration can be estimated. Assuming that each duration corresponded to the time needed by the wave to traverse each segment of the container, both the estimated SWC and the measured SWC at each segment are plotted in Figure 13. Overall, the estimated SWC increased with the measured SWC at each segment, reflecting a linear relationship. For all data, the estimated SWCs were smaller than the measured SWCs. The values of the slope of the linear relationship for the probe lengths of 50 and 100 cm are summarized in Table 4. For dry conditions, the values of the estimated SWC were significantly scattered even in the same segment. Although the linear relationship for all data showed the moderate values of the slope and coefficient of determination, several linear relationships between the estimated and measured SWCs for the first segments in the four treatments showed the higher values of the slope and coefficient of determination.

In practice, the actual length of a partially dry or wet segment along the entire probe length is unknown, and accordingly, the segment length should be estimated. Using the duration and the estimated dielectric constant for each duration, each segment length (*L_ij_*) can be estimated as follows:

(6) $$L_{ij}=\frac{c\left(\Delta t_{ij}\right)}{2\sqrt{{\left(K_{a}\right)}_{ij}}}$$

**Figure 14:**
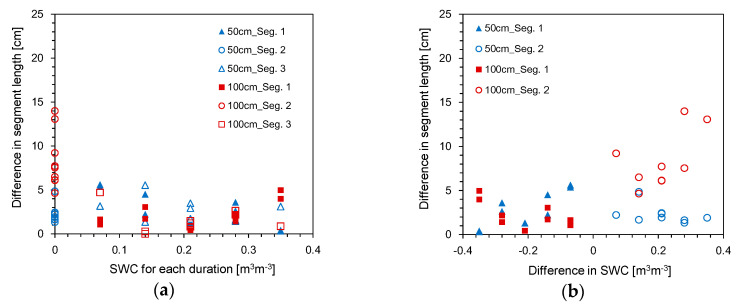
Difference between actual segment length and segment length estimated for each duration in the WDW treatment along; (**a**) SWC; (**b**) difference in SWC.

Figure 14 shows the difference between actual segment length and the segment length estimated for each duration in the WDW treatment. The significant difference in the segment length of 14 cm was found under the dry condition, and except for the dry condition, the difference ranged from 0 to 5.5 cm. The results were in good agreement with those obtained for the comparison of SWCs measured and estimated for dry conditions. Thus, the HETDR could be used to approximately estimate the heterogeneous soil moisture distribution, but the values of SWC and segment length under dry conditions may be significantly underestimated or overestimated. Figure 14b shows that the estimated segment length may be affected by the sequence of soil segments with different water content values and the difference in SWCs between the segments adjacent to each other.

### 4.2. Estimation of Average SWC

**Figure 15:**
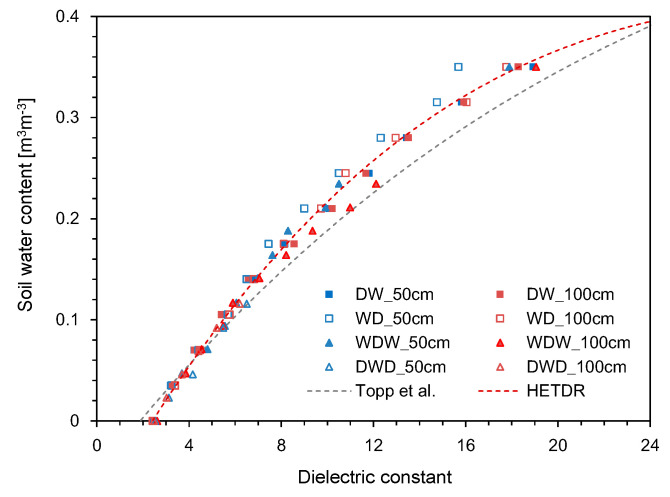
Relationship between the dielectric constant and the SWC.

Using the HETDR system, the dielectric constants were determined based on the travel time of the final reflection as determined from the four treatments. The variations of the average dielectric constant with the average SWC are plotted in Figure 15: This constant increased with the average SWC, reflecting a well-established third degree polynomial relationship (coefficient of determination = 0.993). The relationship between the dielectric constant and the water content can be represented as follows:

(7) $$\theta =1.0\times 10^{-5}K_{a}{}^{3}-1.08\times 10^{-3}K_{a}{}^{2}+4.07\times 10^{-2}K_{a}-9.22\times 10^{-2}\;(R^{2}\;=\;0.9926)$$

where *θ* is the average volumetric water content of the soils. All data plotted in Figure 15 include the results obtained by using probes with lengths of 50 and 100 cm, but the probe length should not affect the accuracy of water content determination for non-conducting media [30]. Compared to the empirical relationship suggested by Topp et al. [12], the dielectric constants determined in this study through the HETDR system for SWC > 0.04 m^3^ m^−3^ were slightly smaller.

**Figure 16:**
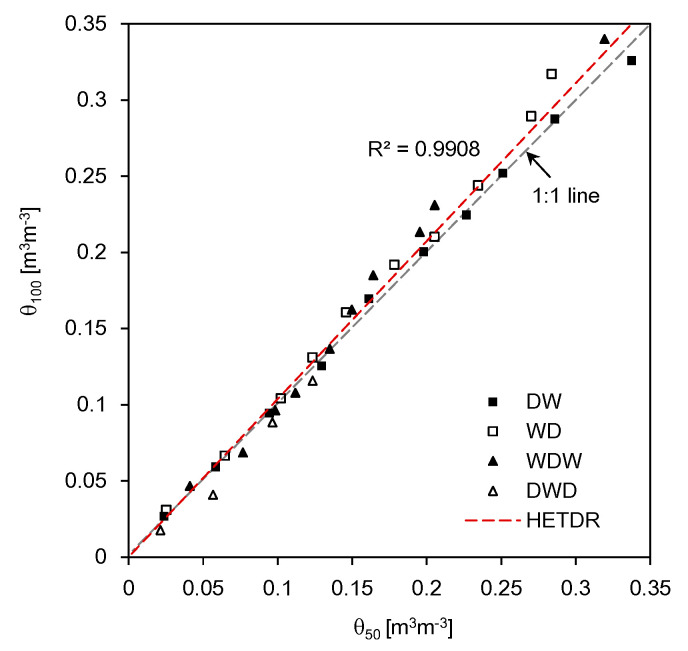
Relationship between the SWCs estimated using probes with lengths of 50 cm and 100 cm. θ_50_ and θ_100_ denote the SWCs estimated from the probes with lengths of 50 cm and 100 cm, respectively.

The average SWCs estimated from the dielectric constants through the four different model tests are plotted in Figure 16. The SWCs estimated by using the HETDR probes with lengths of 50 and 100 cm were almost identical and showed a linear relationship (coefficient of determination = 0.991). At higher water content values, the HETDR probe with a length of 100 cm produced slightly higher values compared to that with a length of 50 cm. Notably, the third degree polynomial relationships established from the calibration led to higher standard deviations for higher water content values.

### 4.3. HETDR versus Conventional TDR

**Figure 17:**
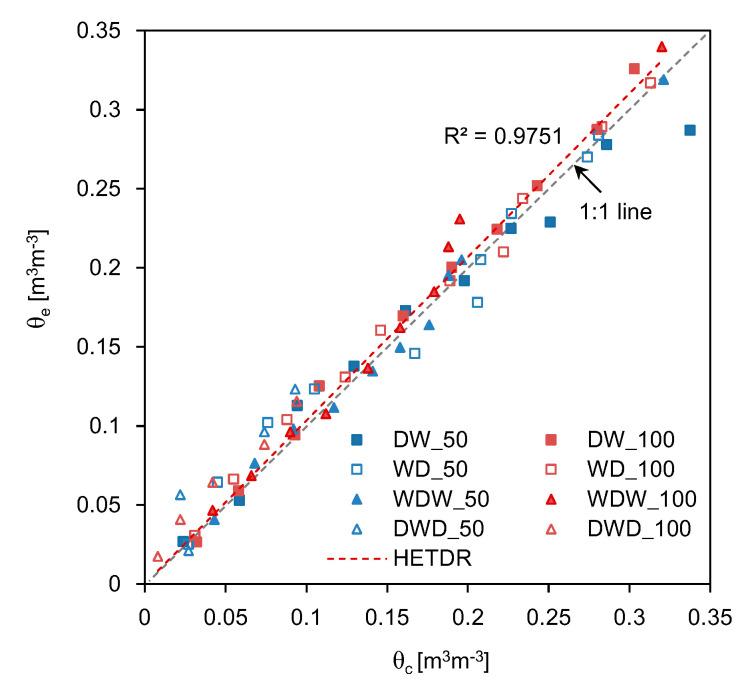
Relationship between the SWCs estimated through the HETDR and conventional TDR probes. θ_e_ and θ_c_ denote the SWCs estimated from HETDR and conventional TDR probes, respectively.

In addition to the HETDR probe, the conventional TDR probe was also used to evaluate water content distributions through four different model tests. The conventional TDR probes were inserted vertically onto the surface of the soil specimens every 10 cm. The average SWCs for the entire segment were estimated by using the conventional TDR probe and compared to those estimated through the HETDR probe (Figure 17). Overall, these values were similar, regardless of the probe length. Since the lengths of HETDR probes were higher than that of the conventional TDR probe, attenuation and dispersion may be more obvious in the case of the HETDR probe, causing a significant increase in the rising time [28]. Consequently, the travel time, dielectric constant, and SWC obtained through the HETDR probe were higher than those obtained through the conventional TDR probe. In addition, when positioning the conventional TDR probe into the soil, an air gap can remain between the probe and the soil, leading to an underestimation of the dielectric constant [23]. A linear relationship was identified between the results of the HETDR and conventional TDR (coefficient of determination = 0.975). Notably, at higher water content values, the values of SWC obtained though the HETDR probe were slightly higher than those of the conventional TDR probe. Moreover, the measuring areas of the HETDR probes were different from those of conventional TDR probes, due to their direction of installation. Considering the strong relationship between the results of the conventional TDR and HETDR probes, the HETDR probes can be effectively used to evaluate the average SWC in the horizontal direction by using a minimal number of probes.

## 5. Conclusions

Soil water content (SWC) is a key factor to consider for establishing effective irrigation plans allowing for the management of water resources. However, most of the existing methods for the measurement of SWC are point-based and vertically applied on the soils. The HETDR system was developed to evaluate the variability of the TDR response, the potential for the estimation of heterogeneous SWC distribution, and the reliability of the average water content estimated for nonuniformly wetted soils. The HETDR probes were calibrated by testing three different probe lengths and spacings in a homogeneous soil with different water content values. The HETDR probes were used in four different treatments for the determination of nonuniform water content distribution. The correspondent results were compared with those of the conventional TDR probe.

The calibration curves of the HETDR probes reflected third degree polynomial relationships, regardless of their different lengths and spacings. For the probes with the same spacing and (three) different lengths, the standard deviations at high SWCs were greater than those at the low SWCs, while the third degree polynomial relationships obtained for the probes showed high coefficients of determination. In the model tests, as the SWC at a segment increased, the average amplitude decreased and the duration increased. Using the second-degree polynomial relationships based on the average amplitudes, the segment length and the SWC at each segment were estimated and compared with the measured ones. Overall, the estimated SWC increased with the measured SWC, and the difference between actual segment length and the segment length estimated from the HETDR probes was significant under dry conditions. Regardless of the water distribution, the average SWCs determined through the two HETDR probes with different lengths were almost identical. Furthermore, the average SWCs estimated through the HETDR probes were quite similar to those estimated through the conventional TDR probe. Therefore, the HETDR system can be effectively used to evaluate average SWCs in the horizontal direction.

## Figures and Tables

**Table 1:** Dimensions of the HETDR probe.

No.	Length, L (cm)	Spacing, s (cm)	Width, D (cm)
1		2.5	5
2	50	5.0	10
3		7.5	15
4		2.5	5
5	75	5.0	10
6		7.5	15
7		2.5	5
8	100	5.0	10
9		7.5	15

**Table 2:** Water content in the soil container divided into different segments.

Treatment	Average	Two Segments		Treatment	Average	Three Segments		
	SWC	First	Second		SWC	First	Second	Third
Dry-Wet (DW)	0	0	0	Wet-Dry-Wet (WDW)	0	0	0	0
	0.035	0	0.07		0.047	0.07	0	0.07
	0.070	0	0.14		0.070	0.07	0	0.14
	0.105	0	0.21		0.093	0.14	0	0.14
	0.140	0.07	0.21		0.117	0.14	0	0.21
	0.175	0.14	0.21		0.140	0.21	0	0.21
	0.210	0.14	0.28		0.163	0.28	0	0.21
	0.245	0.21	0.28		0.187	0.28	0	0.28
	0.280	0.21	0.35		0.210	0.35	0	0.28
	0.315	0.28	0.35		0.233	0.35	0	0.35
	0.350	0.35	0.35		0.350	0.35	0.35	0.35
Wet-Dry (WD)	0	0	0	Dry-Wet-Dry (DWD)	0	0	0	0
	0.035	0.07	0		0.023	0	0.07	0
	0.070	0.14	0		0.047	0	0.14	0
	0.105	0.21	0		0.070	0	0.21	0
	0.140	0.21	0.07		0.093	0	0.28	0
	0.175	0.21	0.14		0.117	0	0.35	0
	0.210	0.28	0.14		0.350	0.35	0.35	0.35
	0.245	0.28	0.21					
	0.280	0.35	0.21					
	0.315	0.35	0.28					
	0.350	0.35	0.35					

Water content values are expressed in m^3^ m^−3^. The head of the HETDR probe was placed at the beginning of the first segment. SWC: soil water content.

**Table 3:** Parameters of dielectric constant and SWC estimated from calibration results for HETDR.

Probe Length	*A*	*B*	*C*	R^2^	*D*	*E*	*F*	R^2^
50 cm	0.0015	0.4021	29.348	0.996	1 × 10^−5^	0.0058	0.5038	0.996
100 cm	0.0022	0.5211	34.566	0.986	1 × 10^−5^	0.0064	0.5766	0.993

**Table 4:** Values of the slope of the linear relationship for SWCs measured and estimated at each segment.

Probe Length		DW		WD		WDW			DWD		
		Seg. 1	Seg. 2	Seg. 1	Seg. 2	Seg. 1	Seg. 2	Seg. 3	Seg. 1	Seg. 2	Seg. 3
50 cm	Slope	0.989	0.646	0.912	0.812	0.908	-	0.594	-	0.550	-
	R^2^	0.993	0.720	0.983	0.887	0.993	-	0.910	-	0.955	-
100 cm	Slope	0.834	0.719	1.006	1.014	1.035	-	0.776	-	0.527	-
	R^2^	0.805	0.723	0.976	0.898	0.962	-	0.918	-	0.918	-
